# The lincRNA *HOTAIRM1*, located in the *HOXA* genomic region, is expressed in acute myeloid leukemia, impacts prognosis in patients in the intermediate-risk cytogenetic category, and is associated with a distinctive microRNA signature

**DOI:** 10.18632/oncotarget.5148

**Published:** 2015-09-11

**Authors:** Marina Díaz-Beyá, Salut Brunet, Josep Nomdedéu, Marta Pratcorona, Anna Cordeiro, David Gallardo, Lourdes Escoda, Mar Tormo, Inmaculada Heras, Josep Maria Ribera, Rafael Duarte, María Paz Queipo de Llano, Joan Bargay, Antonia Sampol, Mertixell Nomdedeu, Ruth M. Risueño, Montserrat Hoyos, Jorge Sierra, Mariano Monzo, Alfons Navarro, Jordi Esteve, on behalf of the Cooperative AML group CETLAM

**Affiliations:** ^1^ Hematology Department, Hospital Clinic, Institut d'Investigacions Bomèdiques August Pi i Sunyer (IDIBAPS), Barcelona, Spain; ^2^ Josep Carreras Leukemia Research Institute (IJC), Barcelona, Spain; ^3^ Hematology Service, Institut d'Investigació Biomèdica Sant Pau, Hospital de la Santa Creu i Sant Pau, Universitat Autònoma de Barcelona, Banc de Sang i Teixits de Catalunya, Spain; ^4^ Laboratory of Hematology Service, Institut d'Investigació Biomèdica Sant Pau. Hospital de la Santa Creu i Sant Pau, Universitat Autònoma de Barcelona, Barcelona, Spain; ^5^ Molecular Oncology and Embryology Laboratory, Human Anatomy Unit, School of Medicine, University of Barcelona, IDIBAPS, Barcelona, Spain; ^6^ Hematology Department, Catalan Institute of Oncology (ICO), Girona, Spain; ^7^ Hematology Department, Hospital Joan XXIII, Tarragona, Spain; ^8^ Hematology Department, Hospital Clínico, Valencia, Spain; ^9^ University Hospital Morales Meseguer, Murcia, Spain; ^10^ Hematology Department, Catalan Institute of Oncology (ICO), Hospital Germans Trias i Pujol, Badalona, Spain; ^11^ Department of Hematology, Catalan Institute of Oncology (ICO), Hospital Duran i Reynals, L'Hospitalet de Llobregat, Barcelona, Spain; ^12^ Hospital Virgen de la Victoria, Málaga, Spain; ^13^ Hospital de Son Llàtzer, Palma de Mallorca, Spain; ^14^ Hospital Son Espases, Palma de Mallorca, Spain; ^15^ University of Barcelona, Barcelona, Spain

**Keywords:** lincRNA, AML, HOTAIRM, HOX, lncRNA

## Abstract

Long non-coding RNAs (lncRNAs) are deregulated in several tumors, although their role in acute myeloid leukemia (AML) is mostly unknown.

We have examined the expression of the lncRNA *HOX antisense intergenic RNA myeloid 1 (HOTAIRM1)* in 241 AML patients. We have correlated *HOTAIRM1* expression with a miRNA expression profile. We have also analyzed the prognostic value of *HOTAIRM1* expression in 215 intermediate-risk AML (IR-AML) patients.

The lowest expression level was observed in acute promyelocytic leukemia (*P* < 0.001) and the highest in t(6;9) AML (*P* = 0.005). In 215 IR-AML patients, high *HOTAIRM1* expression was independently associated with shorter overall survival (OR:2.04;*P* = 0.001), shorter leukemia-free survival (OR:2.56; *P* < 0.001) and a higher cumulative incidence of relapse (OR:1.67; *P* = 0.046). Moreover, *HOTAIRM1* maintained its independent prognostic value within the favorable molecular subgroup (OR: 3.43; *P* = 0.009). Interestingly, *HOTAIRM1* was overexpressed in *NPM1*-mutated AML (*P* < 0.001) and within this group retained its prognostic value (OR: 2.21; *P* = 0.01). Moreover, *HOTAIRM1* expression was associated with a specific 33- microRNA signature that included miR-196b (*P* < 0.001). miR-196b is located in the *HOX* genomic region and has previously been reported to have an independent prognostic value in AML. miR-196b and *HOTAIRM1* in combination as a prognostic factor can classify patients as high-, intermediate-, or low-risk (5-year OS: 24% vs 42% vs 70%; *P* = 0.004).

Determination of *HOTAIRM1* level at diagnosis provided relevant prognostic information in IR-AML and allowed refinement of risk stratification based on common molecular markers. The prognostic information provided by *HOTAIRM1* was strengthened when combined with miR-196b expression. Furthermore, *HOTAIRM1* correlated with a 33-miRNA signature.

## INTRODUCTION

Acute myeloid leukemia (AML) is a highly heterogeneous disease, with diverse biological, phenotypic and prognostic behaviors and strikingly different outcomes to standard therapy. This prognostic diversity is most evident in the cytogenetic intermediate-risk AML subgroup (IR-AML), which is now commonly subdivided into molecular prognostic categories according to the mutational status of *NPM1*, internal tandem duplication of *FLT3* (*FLT3*-ITD), and *CEBPA* [1–3]. Although treatment of IR-AML patients is now tailored according to these molecular categories, the prognosis of many patients is still uncertain and the optimal post-remission therapy is unclear, indicating a clear need for additional prognostic markers.

In recent years, several studies of prognostic markers have focused on the role of RNA molecules that lack protein coding potential, known as non-coding RNAs (ncRNAs). Until recently, the majority of studies of ncRNAs in AML focused on microRNAs (miRNAs) and their roles in pathogenesis and prognosis [4–7], while long non-coding RNAs (lncRNAs, > 200 nt) have not been extensively studied. However, a recent study profiling lncRNAs found specific expression patterns associated with different AML categories and identified a 48-lncRNA score with prognostic implications in a subset of older patients with normal karyotype AML [8].

*HOX* genes play a key role in hematopoiesis [9] and leukemogenesis [10–13] and their expression level has been associated with prognosis in some AML subtypes [14–16]. Maintenance of *HOX* expression patterns is under complex epigenetic regulation and numerous ncRNAs, including lncRNAs, may participate in the regulation of *HOX* expression [17]. For example, *HOTAIR*, a lncRNA located in the genomic region of *HOX*C, regulates the expression of several *HOX* genes [17]. *HOTAIR* cooperates with miR-196a (also located in the *HOX* genomic regions) to drive malignancy in gastric tumors [18]. The lncRNA *HOTTIP*, located in the region adjacent to the *HOXA* locus, is essential for the coordination of several 5′ *HOXA* genes [19] and the expression levels of *HOTTIP* and *HOXA13* have been associated with progression in hepatocarcinoma [20].

lncRNAs transcribed in the intergenic regions, known as long intergenic non-coding RNAs (lincRNAs) [21], are dynamically expressed in hematopoiesis [22] and cancer [23]. *HOTAIRM1* (*HOX antisense intergenic RNA myeloid 1*) is a lincRNA located in the *HOXA* genomic cluster, on chromosome band 7p15 between *HOXA1* and *HOXA2*. *HOTAIRM1* is transcribed antisense by RNA polymerase II. *HOTAIRM1* expression is induced by retinoic acid and modulates the expression of genes involved in myeloid differentiation. It interacts with proteins of the Polycomb repressive complex2 in mouse embryonic stem cells [24]. *HOTAIRM1* is overexpressed in myeloid leukemia cell lines and mature leukocytes compared to hematopoietic stem and progenitor cells [25]. However, *HOTAIRM1* expression and its prognostic impact in AML patients have not yet been investigated.

In the present study, we have analyzed *HOTAIRM1* expression in a large cohort of AML patients (*n* = 241) with several cytogenetic subtypes. We have also explored its potential prognostic value in a subset of 215 IR-AML patients included in intensive therapy protocols for younger patients. Finally, we have examined the potential association between *HOTAIRM1* and a miRNA profile.

## RESULTS

### *HOTAIRM1* expression and clinical, cytogenetic and molecular characteristics

**Figure 1:**
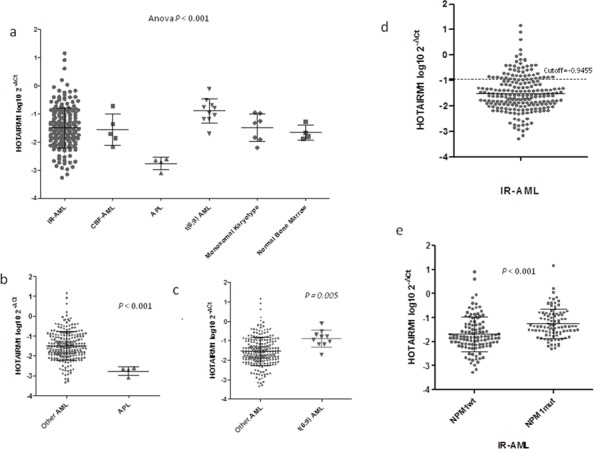
HOTAIRM1 expression levels in AML patients and healthy controls **a.** Expression levels of *HOTAIRM1* varied among the five AML cytogenetic subtypes included in the study, namely cytogenetic intermediate-risk AML (IR-AML), AML with core-binding factor rearrangement (CBF-AML), Acute Promyelocytic leukemia (APL), AML with translocation t(6;9)(p23;q34)/DEK-NUP214 (t(6;9) AML), and monosomal karyotype AML. **b.** *HOTAIRM1* expression was lower in APL than in the other AML subtypes. **c.** *HOTAIRM1* expression was higher in t(6;9) AML than in the other AML subtypes. **d.** The range of HOTAIRM1 expression levels among IR-AML samples, with the cutoff value identified by MaxStat package of R software as having the maximum prognostic impact. **e.** Within the IR-AML subtype, HOTAIRM1 expression was higher in patients with NPM1 mutations.

A significant difference in *HOTAIRM1* expression level was observed among the five AML genetic subgroups included in the study and the healthy controls (ANOVA *P* < 0.001) (Figure 1a). The lowest *HOTAIRM1* levels were observed in APL patients (*P* < 0.001) (Figure 1b), whereas the highest levels were in patients with t(6;9) AML (*P* = 0.005) (Figure 1c). *HOTAIRM1* expression levels were diverse in IR-AML patients (median, −1.49; range, −3.27 to 1.16). This information is shown in Figure 1d. Among IR-AML patients the highest levels were observed in those harboring *NPM1* mutations (*NPM1*mut patients) (*P* < 0.001) (Figure 1e). *HOTAIRM1* expression was not significantly associated with any other mutations (DNMT3A, IDH1 or IDH2) or any clinical features, including age, WBC, bone marrow blast proportion, or FAB subtype.

We validated these results using arrays from the Leukemia-gene Atlas repository (http://www.leukemia-gene-atlas.org/LGAtlas), an *in silico* analysis of *HOTAIRM1* levels in different AML subgroups showed that in other cohorts [26, 27], *HOTAIRM1* was also expressed at the lowest levels in APL and at the highest in t(6;9) AML (Supplementary Figure S1a). Similar to our findings, *HOTAIRM1* expression was also higher in *NPM1*mut patients (Supplementary Figures S1b–S1c).

### Higher *HOTAIRM1* expression associated with worse prognosis in IR-AML

**Figure 2:**
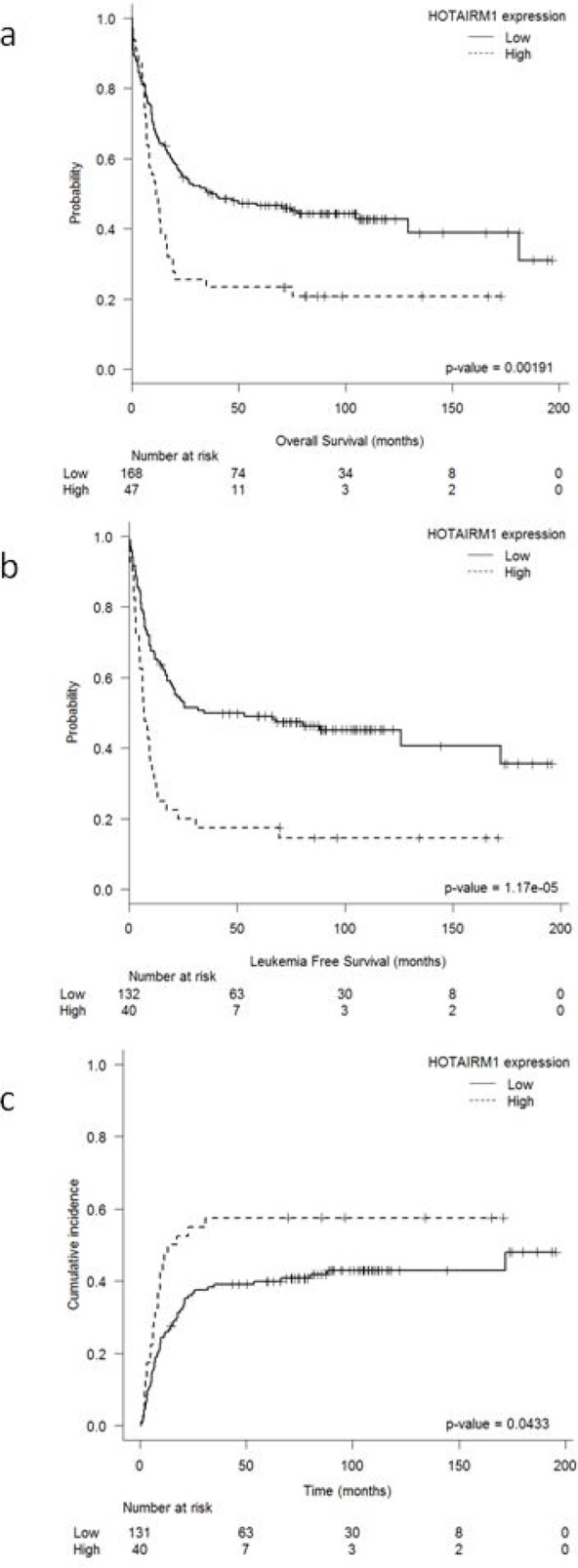
Outcome according to HOTAIRM1 expression levels in IR-AML High *HOTAIRM1* expression was associated with poorer outcome in 215 IR-AML patients, with **a.** shorter overall survival, **b.** shorter leukemia-free survival, and **c.** a higher cumulative incidence of relapse.

With a median follow-up of 7.6 years (range: 15–196 months) among patients alive at last follow-up, the 215 IR-AML patients had a CR rate of 80% and a 5-year OS, LFS, and CIR of 42 ± 6%, 42 ± 7%, and 44 ± 7%, respectively. Figure 1d shows the optimal cutoff level for *HOTAIRM1* expression within the IR-AML as identified by the MaxStat package. Patients with higher *HOTAIRM1* levels had shorter OS and LFS (5-year OS: 23 ± 11% vs. 46 ± 7%, *P* = 0.001; LFS: 17 ± 12% vs.49 ± 8%, *P* < 0.001), and a higher CIR (57 ± 13% vs. 39 ± 8%, *P* = 0.043) compared to patients with lower levels (Figures 2a–2c). *HOTAIRM1* expression levels were not associated with the probability of attaining CR. Moreover, the frequency of allogeneic hematopoietic stem-cell transplantations (alloHSCT) performed in first CR (CR1) did not differ according to *HOTAIRM1* expression levels (21% vs. 20.8% in patients with high and low *HOTAIRM1* levels, respectively, *p* = 0.99).

The multivariate analysis confirmed high *HOTAIRM1* expression as an independent prognostic factor for OS (OR: 2.04; 95%CI:1.36–3.07; *P* = 0.001), LFS (OR: 2.56; 95%CI:1.67–3.92; *P* < 0.001) and CIR (OR:1.67; 95%CI:1.01–2.77; *P* = 0.04), in addition to age (OS, LFS), gender (LFS), WBC count at diagnosis (OS), presence of *FLT3*-ITD (OS, LFS, CIR), and *NPM1* mutations (OS, LFS, CIR) (Table 1).

**Table 1:** Multivariate analyses for overall survival, leukemia-free survival, and cumulative incidence of relapse in the overall series, in patients with *NPM1* mutations (*NPM1*mut patients), and in the favorable molecular category (i.e., *NPM1* mutation without concomitant *FLT3*-ITD or *CEBPA* double mutation), within the cytogenetic intermediate-risk cohort. Age was analyzed with 10-year intervals and white blood cell count at diagnosis using 50 × 10^9^/L increments

Variables	*P*	OR	95%CI	*P*	OR	95%CI	*P*	OR	95%CI
Overall Survival									
	All patients			*NPM1*mut AML			Favorable molecular category		
**Age**	<0.001	1.56	1.34–1.83	<0.001	1.83	1.36–2.48	0.02	1.60	1.07–2.39
**Sex (male vs. female)**	0.076	1.38	0.96–1.99	0.3			0.6		
**WBC**	0.008	1.17	1.03–1.32	0.01	1.26	1.05–1.50	0.01	1.49	1.10–2.01
***FLT3*-ITD**	0.002	1.88	1.27–2.79	0.01	2.07	1.13–3.81			
***NPM1* mutations**	<0.001	0.45	0.31–0.67						
***HOTAIRM1* levels (high vs. low)**	0.001	2.04	1.36–3.07	0.01	2.21	1.18–4.16	0.009	3.43	1.36–8.61
**Leukemia-Free Survival**									
	**All patients**			***NPM1*mut AML**			**Favorable molecular category**		
**Age**	0.001	1.31	1.11–1.53	0.008	1.44	1.10–1.89	0.53		
**Sex (male vs. female)**	0.024	1.57	1.06–2.33	0.5			0.92		
**WBC**	0.2	1.08	0.95–1.23	0.19			0.08		
***FLT3*-ITD**	0.05	1.55	1.00–2.41	0.08	1.7	0.29–3.10			
***NPM1* mutations**	0.001	0.48	0.31–0.73						
***HOTAIRM1* levels (high vs. low)**	<0.001	2.56	1.67–3.92	0.001	2.84	1.51–5.34	0.001	4.64	1.93–11.16
**Cumulative Incidence of Relapse**									
**All patients**				***NPM1*mut AML**			**Favorable molecular category**		
**Age**	0.28			0.07			0.06		
**Sex (male vs. female)**	0.12			0.9			0.47		
**WBC**	0.42			0.48			0.8		
***FLT3*-ITD**	0.02	1.72	1.06–2.78	0.0042	2.07	1.02–4.17			
***NPM1* mutations**	0.004	0.32	0.005–0.81						
***HOTAIRM1* levels (high vs. low)**	0.046	1.67	1.01–2.77	0.011	2.58	1.24–5.33	0.008	3.86	1.40–10.6

We validated our findings on the prognostic value of *HOTAIRM1* expression in another patient population [28] by performing an *in silico* re-analysis of the array data, available in the Leukemia-gene Atlas repository (http://www.leukemia-gene-atlas.org/LGAtlas; Supplementary Figure S1d)

### Higher *HOTAIRM1* expression associated with worse prognosis in *NPM1*mut patients

**Figure 3:**
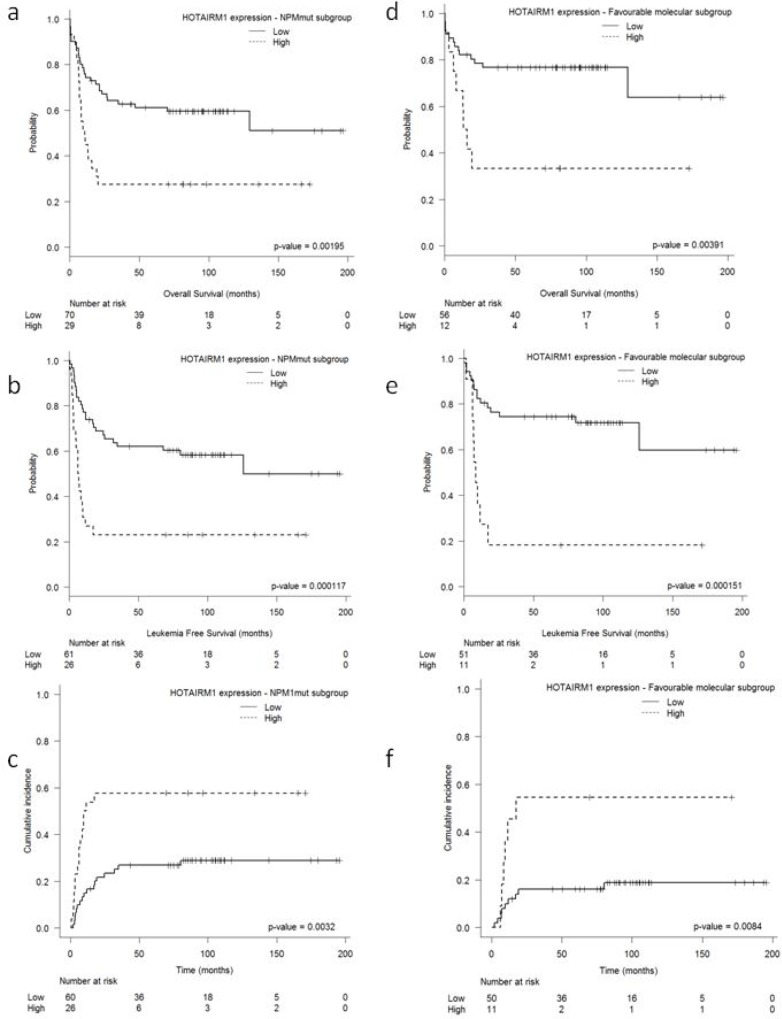
Outcome according to HOTAIRM1 expression levels in (a–c) NPM1mut IR-AML patients and (d–f) patients in the FAVmol subgroup of IR-AML patients **a.** overall survival, **b.** leukemia-free survival, and **c.** cumulative incidence of relapse in *NPM1*mut patients. **d.** overall survival, **e.** leukemia-free survival, and **f.** cumulative incidence of relapse in patients in the FAVmol subgroup.

Since *HOTAIRM1* levels were significantly higher in *NPM1*mut patients (Figure 1e), we analyzed the prognostic value *of HOTAIRM1* in this subset of patients. Since *FLT3*-ITD is one of the most frequent mutations associated with *NPM1* and its presence modifies the prognostic significance of *NPM1,* we compared *HOTAIRM1* expression in patients with only *NPM1* mutations with those with both *NPM1* and *FLT3-ITD* mutations and we did not find any significant difference between both subgroups. Thus, the median value of *HOTAIRM1* expression in patients with concomitant *FLT3-ITD* mutations was 1.32, and 1.18 in patients without *FLT3-ITD* mutations (*p* = 0.12). Among the 99 *NPM1*mut patients, those with high levels of *HOTAIRM1* had shorter OS (5-year OS: 27 ± 14% vs. 61 ± 27%; *P* = 0.001) and LFS (5-year LFS: 22 ± 16% vs. 62 ± 12%; *P* < 0.001) and a higher CIR (5-year CIR: 58 ± 12% vs. 28 ± 9%; *P* = 0.003) (Figure 3a–3c). In the multivariate analyses including only *NPM1*mut patients, *HOTAIRM1* expression retained its prognostic value for OS (OR: 2.21; 95%CI: 1.18–4.16; *P* = 0.014), LFS (OR: 2.84; 95% CI: 1.51–5.34; *P* = 0.001) and CIR (OR: 2.578; 95% CI: 1.245–5.33; *P* = 0.01) (Table 1). Other independent prognostic factors for the *NPM1*mut patients were age (OS, LFS, trend for CIR), WBC at diagnosis (OS), and *FLT3*-ITD (OS, CIR, trend for LFS).

### Higher *HOTAIRM1* expression associated with worse prognosis in the FAVmol subgroup

When we analyzed the specific impact of *HOTAIRM1* expression on outcome in the molecularly defined subgroups (FAVmol and UNFAVmol), among the 68 patients in the FAVmol subgroup, high *HOTAIRM1* expression identified a subset of patients with shorter OS (5-yr OS: 33 ± 20% vs.77 ± 10%; *P* = 0.004) and LFS (5-year LFS:18 ± 20% vs.74 ± 11%; *P* < 0.001) and a higher CIR (5-yr CIR: 54 ± 13% vs. 16 ± 10%; *P* = 0.008) (Figures 3d–3f). In the multivariate analyses, *HOTAIRM1* expression retained its prognostic value in the FAVmol subgroup for OS (OR: 3.43; 95%CI: 1.37–8.61; *P* = 0.009), LFS (OR: 4.64; 95%CI: 1.93–11.1; *P* = 0.001), and CIR (OR: 3.863; 95%CI: 1.41–10.6; *P* = 0.008) (Table 1).

Among the 147 patients in the UNFAVmol subgroup, high levels of *HOTAIRM1* were associated with shorter LFS (5-year LFS: 17 ± 13% vs. 31 ± 10%; *P* = 0.014) (Supplementary Figure S2) but not with OS or CIR.

### Correlation of *HOTAIRM1* with miRNA expression

Based on our previous comprehensive analysis of miRNA expression in 85 patients of the present series [5], we were able to identify a 33-miRNA signature which correlated with *HOTAIRM1* expression (Table 2). This signature included the overexpression of several miRNAs located within the genomic regions of *HOX* gene clusters, including miR-196b (*P* < 0.001, correlation coefficient [CC] = 0.41), miR-10a (*P* = 0.001, CC = 0.33), and miR-10a* (*P* = 0.003, CC = 0.35). In addition, the signature included other miRNAs involved in hematopoiesis and leukemogenesis, such as miR-9 (*P* = 0.005, CC = 0.31), miR-222 (*P* = 0.005, CC = −0.32) and miR-424 (*P* = 0.009, CC = −0.30) (Table 2).

**Table 2:** Thirty-three miRNAs whose expression correlated with *HOTAIRM1* expression

Correlation coefficient	Parametric *p*-value	miRNA
0.412	<0.001	hsa-miR-196b
0.37	0.001	hsa-miR-27a*
0.364	0.001	hsa-miR-34a*
0.3662	0.001	hsa-miR-15b*
0.353	0.001	hsa-miR-10a*
0.347	0.002	hsa-miR-100*
0.342	0.002	hsa-miR-604
0.34	0.002	hsa-miR-641
0.338	0.002	hsa-miR-596
0.333	0.003	hsa-miR-487b
0.333	0.003	hsa-miR-519b
0.333	0.003	hsa-miR-10a
0.33	0.003	hsa-miR-339–5p
0.322	0.004	hsa-miR-188–5p
−0.319	0.005	hsa-miR-222
0.319	0.005	hsa-miR-606
0.316	0.005	hsa-miR-9
0.315	0.005	hsa-miR-519a
0.314	0.005	hsa-miR-520c-3p
0.308	0.006	hsa-miR-610
0.302	0.008	hsa-miR-9*
0.302	0.008	hsa-miR-650
0.301	0.008	hsa-miR-939
0.299	0.008	hsa-miR-580
0.299	0.008	hsa-miR-661
−0.299	0.008	hsa-miR-450b-5p
0.299	0.008	hsa-miR-760
0.299	0.009	hsa-miR-138–1*
0.298	0.009	hsa-miR-433
−0.297	0.009	hsa-miR-424
0.297	0.009	hsa-miR-155*
0.297	0.009	hsa-miR-605
0.297	0.009	hsa-miR-877

**Figure 4:**
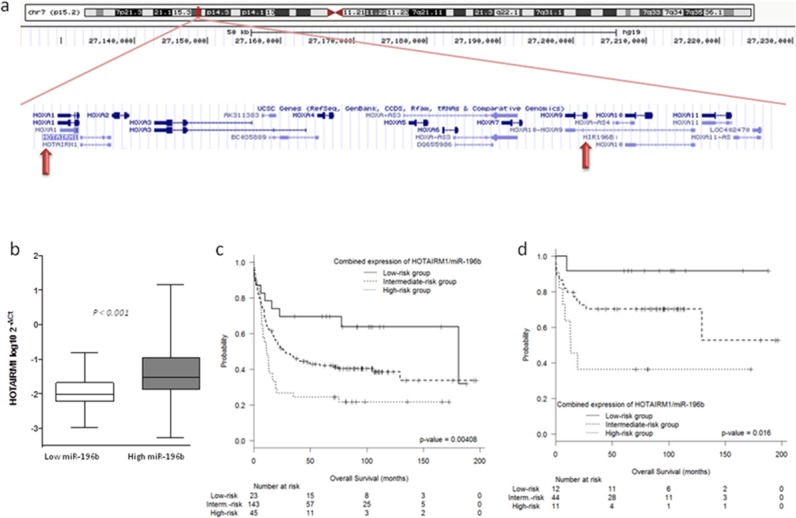
(a–b) Correlation between HOTAIRM1 and miR-196b. (c–d) Prognostic value of the combined expression of HOTAIRM1 and miR-196b **a.** Genomic location of *HOTAIRM1* and miR-196b at the Chr7(p15.2). **b.** Levels of *HOTAIRM1* according to miR-196b expression. **c–d.** Overall survival according to the risk score based on the expression of both *HOTAIRM1* and miR-196b (c) in 215 IR-AML patients and (d) in patients in the FAVmol subgroup.

miR-196b, which showed the most significant association with *HOTAIRM1*, is located in the distal region of the same *HOXA* genomic region (Figure 4a). We therefore analyzed the correlation between miR-196b and *HOTAIRM1* in the 215 IR-AML patients and found that high levels of *HOTAIRM1* were significantly associated with high levels of miR-196b (*P* < 0.001) (Figure 4b).

### The combination of *HOTAIRM1* and miR-196b expression as a prognostic factor in IR-AML

Given the prognostic value of miR-196b expression identified in our previous work [6], the prognostic value of *HOTAIRM1* expression identified in the present study, and the correlation between miR-196b and *HOTAIRM1*, we performed a multivariate analysis for OS including miR-196b and *HOTAIRM1* expression, as well as other recognized prognostic markers (age, gender, WBC, mutational status of NPM1 and FLT3-ITD). Both miR-196b (OR: 2.36; 95% CI: 1.11–5; *P* = 0.026) and *HOTAIRM1* (OR: 1.84; 95% CI: 1.23–2.78; *P* = 0.003) retained their independent prognostic significance.

We then constructed a risk score based on the effect of these two factors in combination in order to identify prognostic subgroups of IR-AML patients. High miR-196b and high *HOTAIRM1* expression were defined as high-risk factors. Patients were classified into three groups according to the number of high-risk factors: low-risk group, 0 factors; intermediate-risk group, 1 factor; and high-risk group, 2 factors. Five-year OS for the low-, intermediate-, and high-risk groups was 70 ± 19%, 42 ± 8%, and 24 ± 12%, respectively (*P* = 0.004) (Figure 4c). Five-year LFS for the low-, intermediate-, and high-risk groups was 68 ± 20%, 47 ± 8%, and 18 ± 12%, respectively (*P* < 0.001) (Supplementary Figure S3a). A non-significant trend towards an effect on 5-year CIR was also observed (*P* = 0.08) (Supplementary Figure S3b).

Interestingly, this miR-196b/HOTAIRM1 combination showed a prognostic impact in the FAVmol subgroup, with 5-year OS for the low-, intermediate- and high-risk groups of 92 ± 16%, 71 ± 12, and 36 ± 29%, respectively (*P* = 0.016) (Figure 4d). Five-year LFS for the low-, intermediate-, and high-risk groups was 88 ± 20%, 70 ± 14%, and 20 ± 22%, respectively (*P* = 0.001) (Supplementary Figure S3c). No association with CIR was observed (Supplementary Figure S3d).

Among the *NPM1*mut patients, only two were classified as low-risk and were not included in the analysis. However, when high-risk patients were compared with intermediate-risk patients, high-risk patients had a shorter 5-year OS (28 ± 16% vs.60 ± 12%, *P* = 0.003), shorter 5-year LFS (24 ± 16% vs.61 ± 12%, *P* < 0.001), and a higher 5-year CIR (56 ± 19% vs.29 ± 11%, *P* < 0.001) (Supplementary Figures S4a–S4c).

The multivariate analyses for OS, LFS and CIR confirmed the miR-196b/HOTAIRM1 combination as an independent prognostic marker in the entire cohort, in *NPM1*mut patients, and in the FAVmol subgroup (Supplementary Table S1).

## DISCUSSION

The present study, performed in 241 AML patients, has shown that lincRNA *HOTAIRM1*, located in the *HOXA* gene region, is differentially expressed in AML cells depending on the AML subtype. Interestingly, in 215 patients with IR-AML who received intensive chemotherapy, *HOTAIRM1* provided independent prognostic information. High *HOTAIRM1* expression identified a subset of patients with worse outcome both in the overall series of 215 IR-AML patients and within molecular prognostic categories as defined by *NPM1*, *FLT3-ITD* and *CEBPA* mutational status. To the best of our knowledge, this is the first time that a lincRNA has been identified as an independent prognostic factor in a large series of intensively treated IR-AML patients. Our findings suggest that a deregulated expression of *HOTAIRM1* might play a role in AML leukemogenesis and outcome.

Although our study included only 26 non-IR-AML patients, interestingly, *HOTAIRM1* expression differed among the five AML cytogenetic subgroups analyzed in our study, with the lowest levels in APL and the highest in t(6;9) AML. Among IR-AML patients, *HOTAIRM1* expression was significantly higher in *NPM1*mut patients. When we validated these results using arrays from the Leukemia-gene Atlas repository (http://www.leukemia-gene-atlas.org/LGAtlas), an *in silico* analysis of *HOTAIRM1* levels in different AML subgroups showed that in other cohorts [26, 27], *HOTAIRM1* was also expressed at the lowest levels in APL and at the highest in t(6;9) AML (Supplementary Figure S1a). Similar to our findings, *HOTAIRM1* expression was also higher in *NPM1*mut patients (Supplementary Figures S1b–S1c).

*HOX* clusters have a specific pattern of lineage-restricted expression, where *HOXA* genes are predominantly expressed in myeloid cells [29]. Moreover, *HOXA* genes are deregulated in several AML subtypes. *HOXA* gene cluster is downregulated in APL [30], which is in line with our findings on *HOTAIRM1*, and in CEBPA-mutated patients [31]. In contrast, upregulation of some genes of the *HOXA* cluster has been observed in MLL-AML [32], MYST3-CREBBP AML [33], NUP98-fusion gene AML [34, 35], and *NPM1*mut AML [14, 36, 37], which is in line with our results. *HOTAIRM1* expression in our study parallels that reported for some *HOXA* genes in previous studies in APL and *NPM1*mut AML, lending support to the proposal by Sessa et al that the intergenic non-coding transcription of the *HOX* genomic regions may be crucial to maintaining the active state of HOX clusters [38].

In the present study, the highest levels of *HOTAIRM1* expression were found in t(6;9) (p22;q34) DEK/NUP214 AML. *HOXA* gene upregulation has previously been described in *SET-NUP 214* leukemia [29], another *NUP214*-fusion gene leukemia, through binding to *HOXA* promoters. This binding facilitates the recruitment of DOTL1, which in turn activates the transcription of some members of the *HOXA* cluster [39]. Given the similarities between *DEK-NUP214* and *SET-NUP214* fusion proteins, both of which retain the CC and GLFG domains of NUP214 and also exhibit a similar gene-expression profile [39], it is likely that DEK-NUP214 functions in a similar fashion to SET-NUP214, namely by binding to the promoter regions of specific *HOXA* genes, which ultimately results in *HOTAIRM1* overexpression.

Overexpression of *HOTAIRM1* was independently associated with worse outcome in the entire series of 215 IR-AML patients, with shorter OS (*P* = 0.001) and LFS (*P* < 0.001) and higher CIR (*P* = 0.04). Moreover, we confirmed the prognostic value of *HOTAIRM1* expression in patients in the FAVmol subgroup (with *NPM1* or *CEBPA* mutations but without *FLT3*-ITD mutations, defined as favorable in the ELN classification [1]). In addition, since *HOTAIRM1* expression was particularly high in *NPM1*mut patients, we analyzed its prognostic value in these patients and were able to confirm its prognostic value, independently of additional features such as *FLT3*-ITD. Patients in the FAVmol subgroup or *NPM1*mut patients are not usually considered as candidates for alloHSCT in first CR, although approximately one-third of these patients will experience relapse after high-dose cytarabine-based post-remission frontline therapy. The identification of a high-risk subset among these patients could thus be useful in determining post-remission management [40], and the analysis of *HOTAIRM1* expression may well be an additional tool for refining prognosis based on mutational status. Patients identified as being at high risk of relapse based on *HOTAIRM1* expression could then be monitored closely to detect minimal residual disease and could be considered candidates for alloHSCT in first CR if MRD clearance kinetics confirmed this higher risk. In the UNFAVmol subgroup of patients (with *FLT3*-ITD mutations or without *NPM1* or *CEBPA* mutations, defined as intermediate-1 and intermediate-2 in ELN classification [1]), *HOTAIRM1* overexpression was associated with shorter LFS, but with no difference in OS.

We validated our findings on the prognostic value of *HOTAIRM1* expression in another patient population [28] by performing an *in silico* re-analysis of the array data, available in the Leukemia-gene Atlas repository (http://www.leukemia-gene-atlas.org/LGAtlas; Figure S1d). These results are in line with those of a previous analysis of array data in astrocytoma, which showed that *HOTAIRM1* overexpression is associated with a more aggressive grade [41].

A complex regulatory interaction between lncRNAs, mRNAs and miRNAs has been described [42, 43] and lncRNAs seem to regulate both the expression of neighboring genes and distant genomic sequences [44]. Moreover, *HOX* genomic regions have numerous ncRNAs, suggesting that these ncRNAs may participate in the regulation of *HOX* expression [17]. Specifically, *HOTAIRM1* quantitatively impairs expression of *HOXA1* and *HOXA4* [25]. In addition, *HOTAIRM1* regulates cell cycle progression during myeloid maturation in the NB4 human promyelocytic leukemia cell line [45].

We also found that *HOTAIRM1* expression was significantly associated with a 33-miRNA signature. The strongest association was with miR-196b, located in the distal part of the same *HOXA* cluster. Since our group had previously observed that miR-196b expression has prognostic value in IR-AML [6], we included this variable in a multivariate analysis and confirmed that both miR-196b and *HOTAIRM1* retained their independent prognostic significance. Moreover, the combination of *HOTAIRM1*/miR-196b expression yielded a simple risk score which enabled IR-AML patients to be classified in three prognostic groups; in fact, the risk score also had an impact in the FAVmol subgroup. Along these same lines, cooperation between the lncRNA *HOTAIR* and miR-196a, both located within the *HOXC* cluster, has previously been described in gastric cancer, where both had prognostic value and the upregulation of both increased malignancy [18]. Indeed, the putative functional cooperation of the two ncRNAs, miR-196b and *HOTAIRM1*, in normal hematopoiesis and leukemia merits further investigation.

Two other miRNAs in the 33-miRNA signature – miR-10a and miR-10a*, contained within the *HOXB* gene cluster – also correlated with *HOTAIRM1* expression. In addition, the signature includes other miRNAs involved in normal hematopoiesis and deregulated in AML, such as miR-9 [46], miR-222 [47], and miR-424 [47]. Interestingly, miR-424 and *HOTAIRM1* were inversely correlated. Upregulation of miR-424 is involved in monocyte differentiation through miR-424-dependent translational repression of NFI-A [48], while *HOTAIRM*1 is upregulated during myeloid differentiation but not during monocytosis [25]. Finally, the study of the putative pathways regulated by this *HOTAIRM1*-miRNA signature was performed using miR-Path [49] identified signaling pathways deregulated in AML included those signalized by c-KIT and FLT3 (Supplementary Figure S5).

In summary, *HOTAIRM1* expression was observed in a molecular subtype-dependent manner and seems to parallel some *HOXA* expression. Moreover, determination of *HOTAIRM1* level at diagnosis provided relevant prognostic information in a large series of IR-AML patients and allowed refinement of risk stratification based on common molecular markers. This additional prognostic information provided by *HOTAIRM1* expression was strengthened when combined with miR-196b expression. Furthermore, *HOTAIRM1* correlated with a 33-miRNA signature containing several miRNAs with a role in normal hematopoiesis and leukemogenesis, including miR-196b. Taken together, these findings indicate that future functional studies of *HOTAIRM1* are warranted to elucidate its role in AML and its impact on the aggressiveness of the disease.

## MATERIALS AND METHODS

### Patients and treatment

The analysis of *HOTAIRM1* expression was performed in diagnostic samples from 241 patients diagnosed with *de novo* AML from the Cooperative Spanish group CETLAM. According to the MRC classification [2], 215 were IR-AML. The main characteristics of all patients are summarized in Table 3. In addition, mRNA from bone marrow samples from four healthy individuals was included in the study. All patients and controls provided their written informed consent in accordance with the Declaration of Helsinki, and the Ethics Committee of Hospital Clinic of Barcelona approved the study.

**Table 3:** Main clinical characteristics of patients included in the study

***Overall series n* = 245**	
**AML cytogenetic subtypes** Core-binding factor-AML t(8;21)/RUNX1-RUNXT1 inv(16)/t(16;16)/MYH11-CBFbeta AML Acute promyelocytic leukemia t(6;9)AML Monosomal karyotype^1^ AML Intermediate-risk AML **Bone marrow from healthy donors**	5 3 2 4 10 7 215 4
***Intermediate risk AML n = 215***	
**Year of diagnosis** (range)	1994–2009
**Gender** *n* (%) Male Female	114 (53%) 101 (47%)
**Median age, years** (range)	51 (17–71)
**Leukocyte count at diagnosis**, × 10^9^/L median (range)	28 (0.7–408)
**FAB subtype** (n) M0 M1 M2 M4 M5 M6 M7	10 53 38 54 50 8 2
**Cytogenetics** n (%) Normal karyotype Other intermediate-risk	155 (72%) 60 (28%)
**Molecular features** n (%) *NPM1* mutation *FLT3*-ITD *CEBPA* biallelic mutation	99/212 (41%) 75/214 (35%) 17/144 (11%)
**Therapeutic protocol (CETLAM group)** AML-94 AML-99 AML-2003	10 (4%) 31 (14%) 174 (82%)
**Outcome** Complete response to induction regimen Overall survival (5-yr) Leukemia-free survival (5-yr) Cumulative incidence of relapse (5-yr)	86% 42 ± 6% 42 ± 7% 44 ± 7%

1 Defined according to Breems *et al* [53]

All 215 IR-AML patients were treated from 1994 to 2009 in three consecutive CETLAM trials of intensive chemotherapy for fit patients: AML-94 (*n* = 10); AML-99 (*n* = 31) (NCT01716793); AML-03 (*n* = 174) (NCT01723657). Briefly, the induction regimen of AML-94 was ICE (idarubicin, standard-dose cytarabine, and etoposide), while in AML-99 and AML-03 it consisted of one or two courses of IDICE (idarubicin, intermediate-dose cytarabine, and VP-16), with or without priming with G-CSF. All patients achieving complete remission (CR) received an additional course of chemotherapy with mitoxantrone and high-dose cytarabine, and then a transplant decision was made. In protocols AML-99 and AML-03, an autologous hematopoietic stem-cell transplantation (autoHSCT) was planned for patients harboring a normal karyotype without additional risk factors, whereas an allogeneic HSCT (alloHSCT) in first CR (CR1) was recommended for the remaining patients with an available donor. Risk factors considered for risk assignment were the need for two induction courses to achieve CR, detectable minimal residual disease (MRD) by flow cytometry (AML-03), and presence of *FLT3*-ITD (AML-03). In AML-94, post-remission strategy (autoHSCT vs. alloHSCT) depended exclusively on the availability of an HLA-identical sibling.

### RNA extraction

Samples were obtained from bone marrow aspirates in 232 (96%) patients and from peripheral blood, with a minimum blast infiltration of 80%, in the remaining 9 patients. Mononuclear cells were purified by Ficoll density gradient centrifugation and total RNA was isolated using Trizol reagent according to manufacturer's protocol (Invitrogen, Paisley, UK).

### mRNA expression analysis

cDNA was synthesized from 1000 nanograms of total RNA using TaqMan Reverse Transcription Reagent Kit (Applied Biosystems). Real-time PCR was performed in the ABI Prism 7500 Sequence Detection System (Applied Biosystems) using TaqMan Gene expression assays (Applied Biosystems) to determine mRNA levels of *HOTAIRM1* (Hs03296533_g1) and *GUSB*(Hs00939627_m1). All gene expression determinations were run in triplicate. Relative quantification was calculated using the 2^−ΔCt^ method using GUSB as housekeeping gene.

### miRNA quantification

*HOTAIRM1* level was correlated with miRNA expression data obtained in previous studies by our group [5, 6]. Briefly, in these studies, after first performing a comprehensive miRNA expression analysis of 670 mature human miRNAs in tumor samples from 85 IR-AML patients using TaqMan^®^ Array Human MicroRNA Set Cards v2.0 (AB), we selected a group of ten miRNAs with potential prognostic value (miR-644, miR-196b, miR-409-3p, miR-135a, let-7a*, miR-23a*, miR-627, miR-200c, miR-23b and miR-361–3p). These miRNAs were subsequently analyzed by individual assays.

### Molecularly defined prognostic subgroups in IR-AML

The presence or absence of *FLT3*-ITD, *NPM1* and biallelic *CEBPA* mutations have a strong prognostic impact in patients with IR-AML [1, 3]. According to the European LeukemiaNet (ELN) prognostic classification, patients with the *NPM1* mutation or the biallelic *CEBPA* mutation but without the *FLT3*-ITD mutation, when associated to normal cytogenetics, comprise a favorable genetic group – with better prognosis, while patients with the *FLT3*-ITD mutation and/or without the *NPM1* and the biallelic *CEBPA* mutation comprise the intermediate-I and intermediate-II genetic groups. In the present study, we have classified all IR-AML patients with *NPM1* mutations or biallelic *CEBPA* mutations but without *FLT3*-ITD mutations as the favorable molecular (FAVmol) subgroup and all remaining IR-AML patients as the unfavorable molecular (UNFAVmol) subgroup.

### Clinical endpoints and statistical methods

Overall survival (OS) was calculated from diagnosis to death and leukemia-free survival (LFS) from CR to relapse or death. Both OS and LFS were estimated with the Kaplan-Meier method and comparisons among subgroups of patients were performed using the log-rank test. Relapse risk (RR) was calculated from CR to relapse and estimated using the cumulative incidence of relapse (CIR) method computed with the cmprsk package for R 2.12 software. The competing event in the RR analysis was death without relapse. Comparison of RR between groups of patients was performed using the Gray test [50]. Characteristics between groups were compared using the χ^2^ test and Fisher's exact test, when applicable, for categorical variables, and the *t*-test for continuous variables. Multivariate analyses for OS and LFS were performed using the Cox proportional hazards model including age (10-year intervals), gender, white blood cell count (WBC; 50 × 10^9^/L increments) at diagnosis, mutational status of *NPM1* and *FLT3*-ITD, and *HOTAIRM1* expression level. A multivariate analysis for CIR was performed using the subdistribution regression model of Fine and Gray [51] with the cmprsk package. The proportional hazard assumption was tested for each variable by analyzing the Schoenfeld residuals. Kaplan-Meier survival curves were then drawn for *HOTAIRM1* expression predicted to show a survival risk either above or below average risk, using the cutoff points of *HOTAIRM1* expression levels identified by MaxStat package of R software. All analyses were performed with SPSS v.20 or R software version 2.12.2. Significance was set at ≤ 0.05. For the identification of miRNAs significantly correlated with *HOTAIRM1* expression, we used data from our previously identified miRNA profile [5] and a quantitative trail analysis from BRB Array Tools version 3.5.0 [52] with adjustment for multiple comparisons.

## SUPPLEMENTARY FIGURES AND TABLE


