# Supplementary material for: The lincRNA HOTAIRM1, located in the HOXA genomic region, is expressed in acute myeloid leukemia, impacts prognosis in patients in the intermediate-risk cytogenetic category, and is associated with a distinctive microRNA signature
Source: Oncotarget. 2015 Sep 11;6(31):31613–27. doi: 10.18632/oncotarget.5148 (PMC4741628; doi:10.18632/oncotarget.5148)
Supplement: Supplementary file 1 [file oncotarget-06-31613-s001.pdf]

SUPPLEMENTARY FIGURES AND TABLE

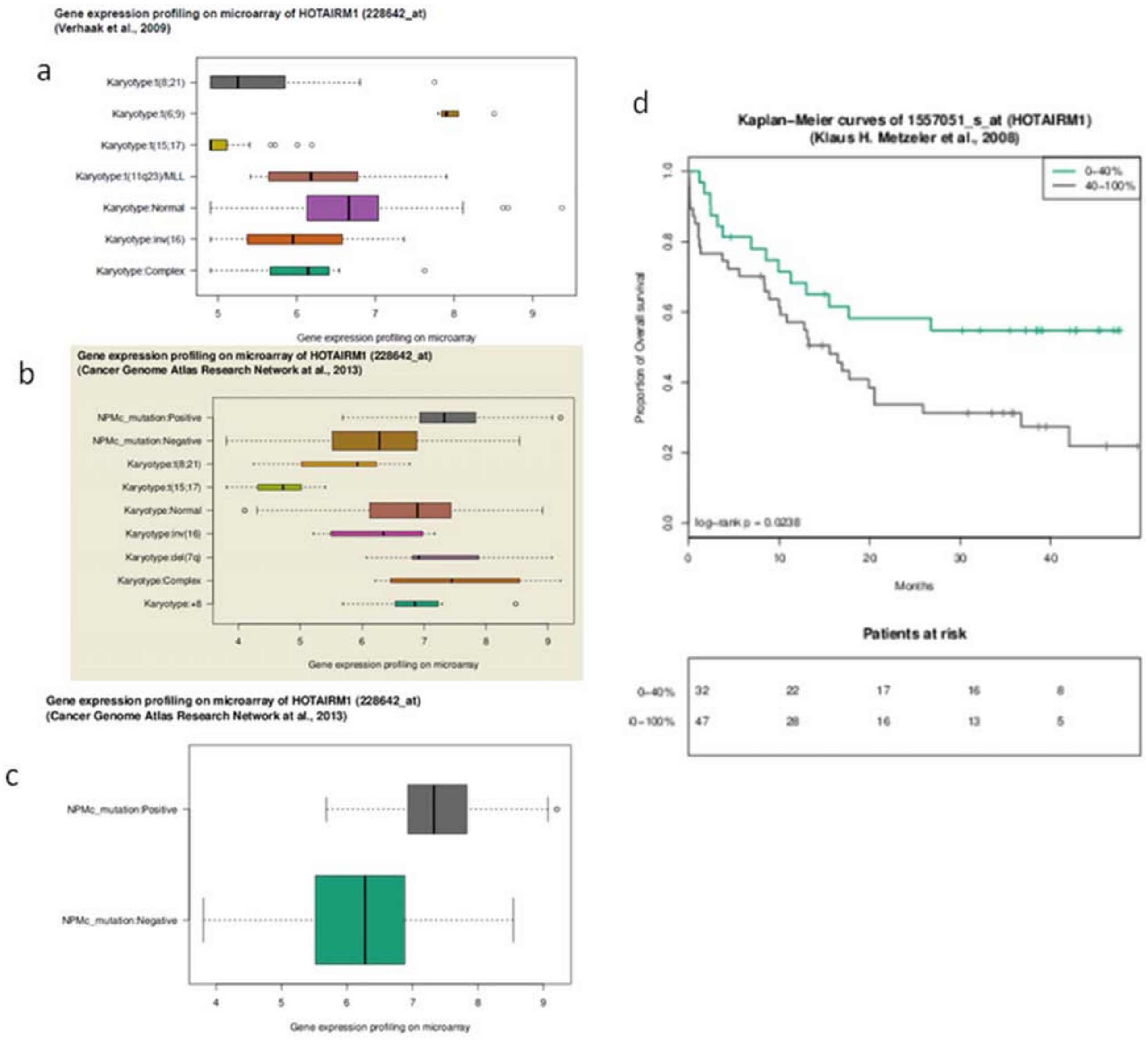

Supplementary Figure S1: *HOTAIRM1* expression levels in different AML cytogenetic/molecular subgroups a–c. Prognostic value of HOTAIRM1 expression.

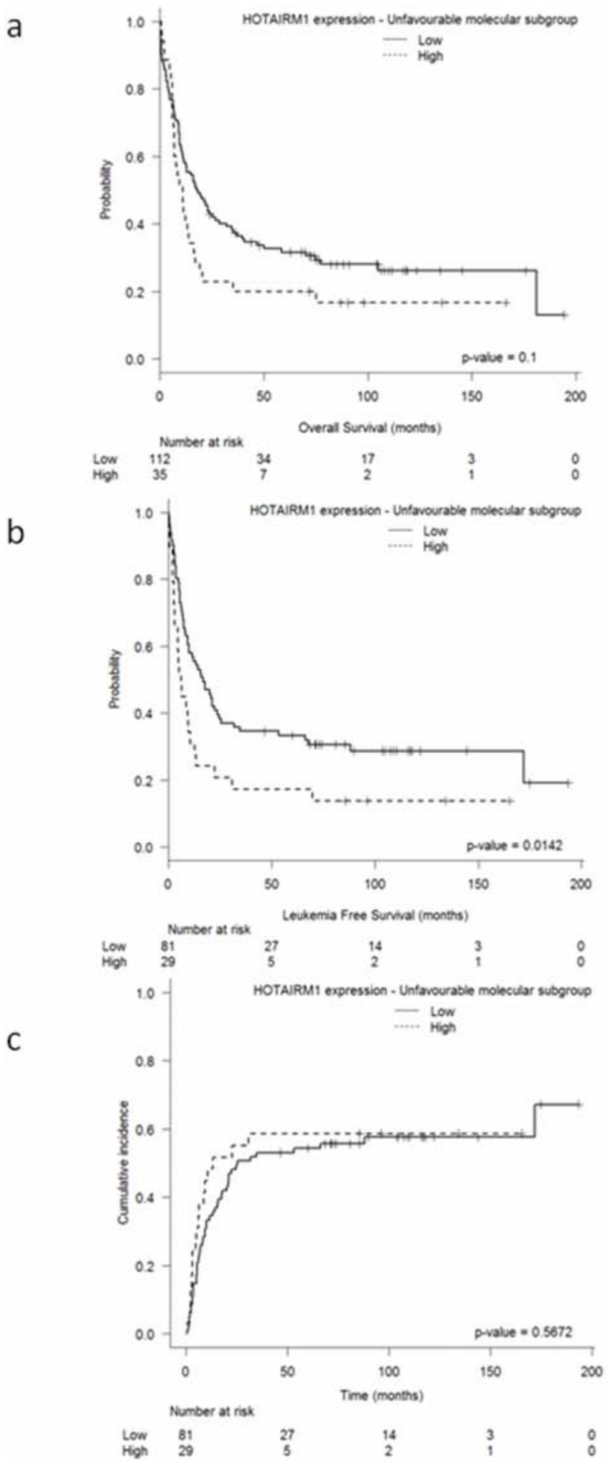

Supplementary Figure S2: a. overall survival, b. leukemia-free survival, and c. cumulative incidence of relapse in patients in the unfavorable molecular subgroup according to levels of *HOTAIRM1* expression.

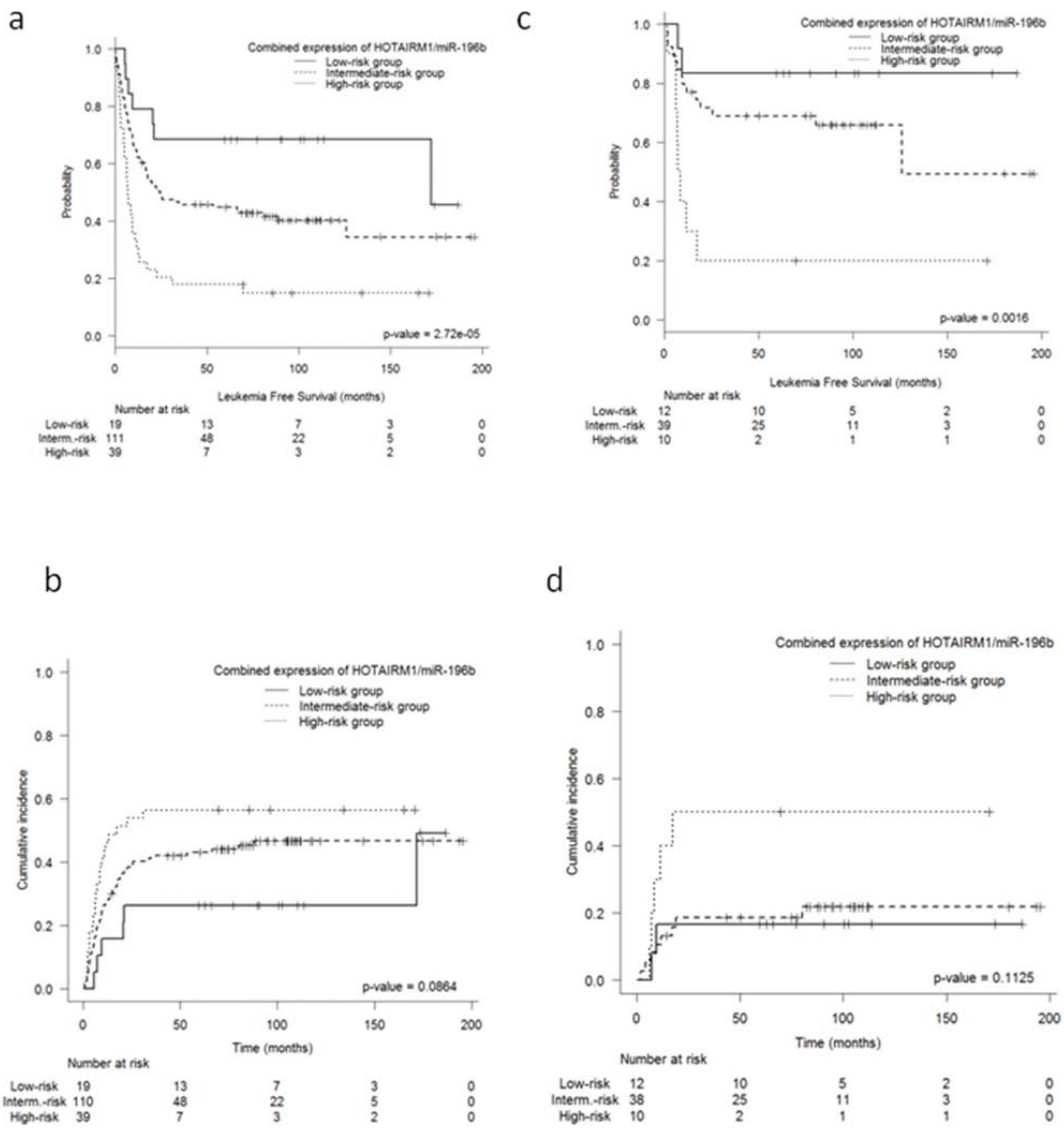

Supplementary Figure S3: a. leukemia-free survival and b. cumulative incidence of relapse in IR-AML patients according to the risk score based on the expression of *HOTAIRM1* and miR-196b. c. leukemia-free survival and **d.** cumulative incidence of relapse in patients in the favorable molecular subgroup according to the risk score based on the expression of HOTAIRM1 and miR-196b.

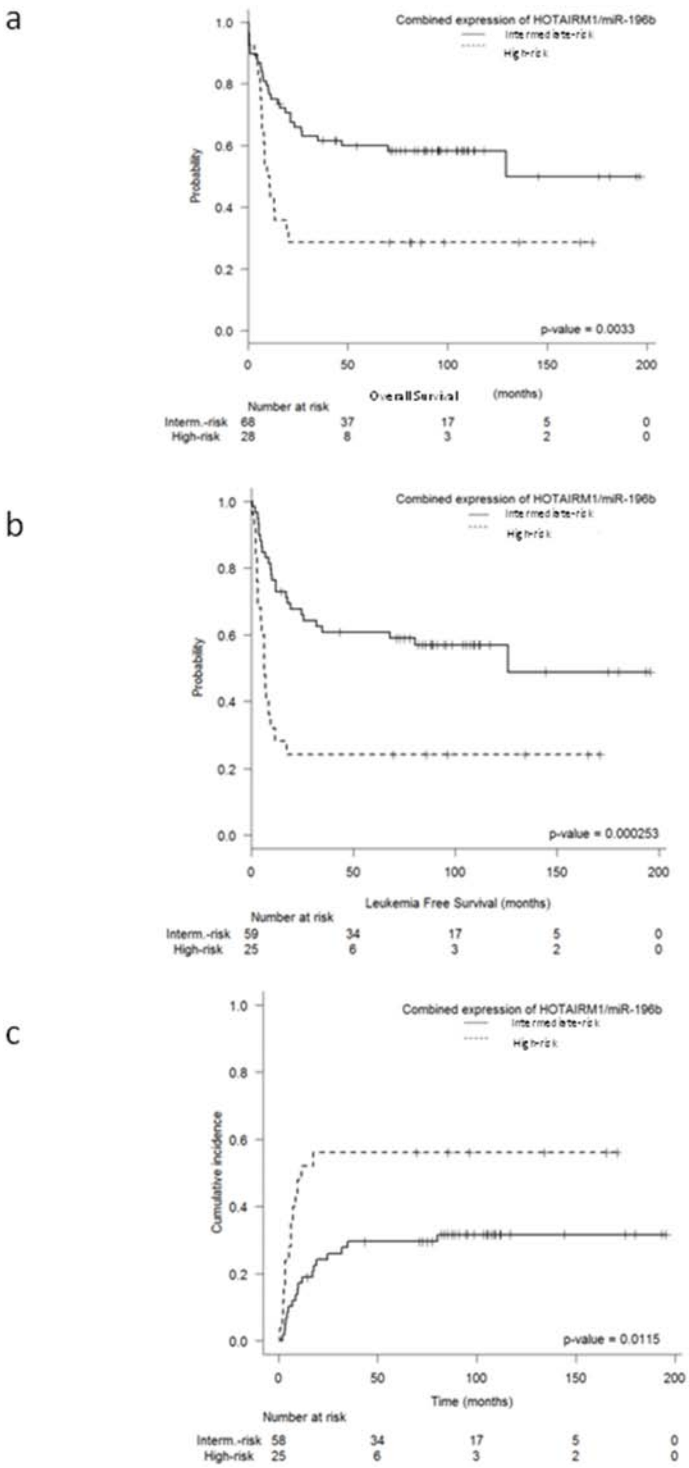

Supplementary Figure S4: a. overall survival, b. leukemia-free survival, and c. cumulative incidence of relapse in *NPM1*mut patients according to the risk score based on the expression of *HOTAIRM1* and miR-196b.

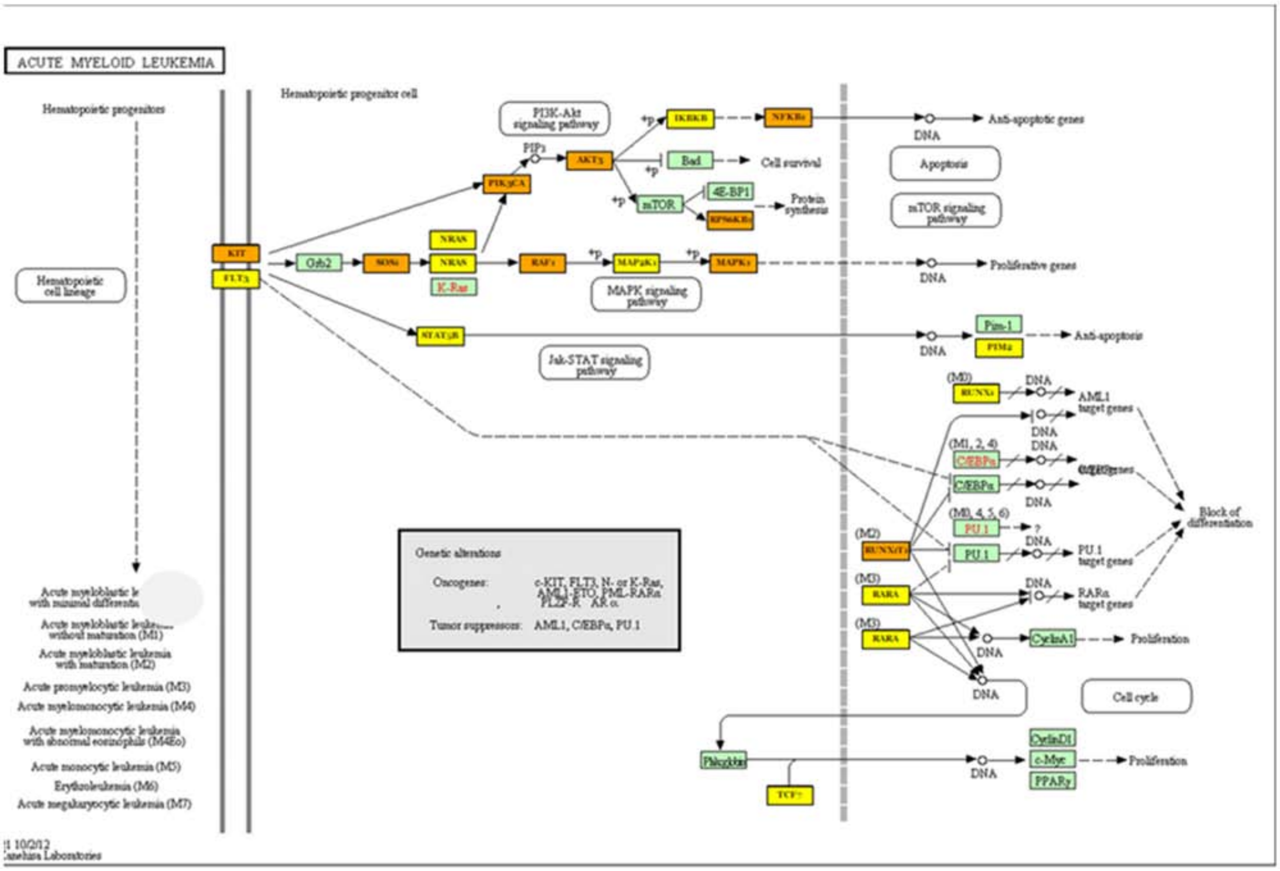

Supplementary Figure S5: Putative pathways regulated by the *HOTAIRM1*-miRNA signature, as identified by miR-Path.

Supplementary Table S1: Multivariate analyses for overall survival, leukemia-free survival, and cumulative incidence of relapse in the overall series, in patients with *NPM1* mutations (*NPM1*mut patients), and in the favorable molecular category (i.e., *NPM1* mutation without concomitant FLT3-ITD or CEBPA double mutation), within the cytogenetic intermediate-risk cohort

Variables	*P*	OR	95%CI	*P*	OR	95%CI	*P*	OR	95%CI
	All Patients			NPM1mut Patients*			FAVmol Subgroup		
				Overall Survival					
**Age**	<0.001	1.54	1.32–1.801	< 0.001	1.79	1.33–2.41	0.033	1.53	1.03–2.25
**Sex**	0.12			0.39			0.54		
**WBC**	0.10	1.17	1.04–1.31	0.01	1.26	1.05–1050	0.024	1.42	1.034–2.25
***FLT3*-ITD**	0.004	1.78	1.21–2.64	0.017	2.095	1.14–3.84			
***NPM1* mutations**	<0.001	0.43	0.29–0.64						
**miR-196b-*HOTAIRM1* risk score**	<0.001	1.90	1.36–2.65	0.053	1.845	0.99–3.43	0.018	2.58	1.17–5.66
				Leukemia-Free Survival					
**Age**	0.002	1.29	1.10–1.51	0.009	1.42	1.10–1.85	0.68		
**Sex**	0.058			0.7			0.96		
**WBC**	0.22			0.19			0.14		
***FLT3*-ITD**	0.076			0.088					
***NPM1* mutations**	<0.001	0.44	0.28–0.67						
**miR-196b-*HOTAIRM1* risk score**	< 0.001	2.31	1.62–3.28	0.006	2.37	1.27–4.41	0.004	3.04	1.47–6.52
				Cumulative Incidence of Relapse					
**Age**	0.41			0.082			0.07		
**Sex**	0.16			1			0.5		
**WBC**	0.43			0.48			0.7		
***FLT3*-ITD**	0.03	1.67	1.03–2.71	0.047	2.03	1.01–4.09			
***NPM1* mutations**	0.003	0.49	0.31–0.79						
**miR-196b-*HOTAIRM1* risk score**	0.01	1.61	1.11–2.3	0.014	2.36	1.20–4.65	0.063		
